# Development and Characterization of Genic SSR Markers from Indian Mulberry Transcriptome and Their Transferability to Related Species of *Moraceae*

**DOI:** 10.1371/journal.pone.0162909

**Published:** 2016-09-26

**Authors:** B. Mathi Thumilan, R. S. Sajeevan, Jyoti Biradar, T. Madhuri, Karaba N. Nataraja, Sheshshayee M. Sreeman

**Affiliations:** Department of Crop Physiology, University of Agricultural Sciences, GKVK Campus, Bengaluru, Karnataka – 560 065, India; National Institute for Plant Genome Research, INDIA

## Abstract

Improving mulberry leaf production with enhanced leaf quality holds the key to sustain the ever increasing demand for silk. Adoption of modern genomic approaches for crop improvement is severely constrained by the lack of sufficient molecular markers in mulberry. Here, we report development and validation of 206 EST derived SSR markers using transcriptome data generated from leaf tissue of a drought tolerant mulberry genotype, Dudia white. Analysis of transcriptome data containing 10169 EST sequences, revealed 1469 sequences with microsatellite repeat motifs. We designed a total of 264 primers to the most appropriate repeat regions, of which 206 were locus specific. These markers were validated with 25 diverse mulberry accessions and their transferability to closely related species belonging to family *Moraceae* was examined. Of these markers, 189 revealed polymorphism with up to 8 allelic forms across mulberry species, genotypes and varieties with a mean of 3.5 alleles per locus. The markers also revealed higher polymorphic information content of 0.824 among the accessions. These markers effectively segregated the species and genotypes and hence, can be used for both diversity analysis and in breeding applications. Around 40% of these markers were transferable to other closely related species. Along with the other genic and genomic markers, we report a set of over 750 co-dominant markers. Using these markers we constructed the first genetic linkage map of mulberry exclusively with co-dominant markers.

## Introduction

India presently is the second largest producer of raw silk next to China [1]. However, the total raw silk produced in India (23000 MT) is far below that produced in China [2][1]. Two major factors emerge as plausible reasons. A cross between multi and bivoltine races of the silk worm (*Bombyx mori* L.) is predominantly reared in India. Though multivoltine races are resilient to tropical climates, cocoon production efficiency is significantly lower than the bivoltine races reared in China [1]. The second and the most important constraint is the low production of mulberry (*Morus* sp.) leaf predominantly due to insufficient water availability. Although significant progress has been made in evolving high yielding cultivars, their productivity severely constrained by water limitations even under irrigated conditions [1][3]. Thus, efforts are being made to improve the crop performance under water limiting conditions. However, because of the perennial and outbreeding nature of mulberry, conventional breeding for a focused crop improvement has been a challenge.

Breeding to improve yield potential, stress resilience and quality of mulberry leaf represents a formidable challenge. Progress in achieving the envisaged goals requires the use of modern breeding approaches utilizing DNA based molecular markers, especially co-dominant marker systems. Among several marker systems available, microsatellite markers or Simple Sequence Repeat (SSR) markers, because of their multi-allelic nature and co-dominant segregation patterns, are the most appropriate to assess diversity among highly cross-pollinated heterozygous genomes like mulberry [4][5][6]. In an earlier study, we reported a large number of genomic SSR markers that can be effectively used for assessing molecular diversity among mulberry accessions [7] which was a significant addition to a very small number of markers available for mulberry until then.

Though the SSR regions are more abundant in non-coding regions of the genomes, expressed regions also may harbor such repeat motifs [8][9]. Polymorphism in the genic regions, though less, has a greater likelihood of identifying functional variability among genotypes. In this paper, we report development of more than 200 genic SSR markers identified by analyzing a set of mulberry transcriptome generated using a germplasm accession of mulberry with superior drought adaptive traits, viz., Dudia white [10].

## Materials and Methods

### Development of EST microsatellite markers

In an earlier study, we generated a large number of Expressed Sequence Tags (ESTs) from the leaf tissue of a mulberry genotype (Dudia white) exposed to drought stress by global transcriptome analysis [10]. The transcriptome data used in this study is available at the National Center for Biotechnology Information’s (NCBI) Sequence Read Archive (SRA) with the study accession number of SRP047446 [10]. A set of 10,169 EST sequences formed the basis for the development of genic SSR markers.

Initially, the EST sequences were subjected to CD-HIT analysis (http://weizhongli-lab.org/cd-hit/) to identify unique and non-redundant sequences for designing primers. The nucleotide sequences were analyzed using the Clustal-W tool [11] to determine the complementarities between pairs of sequences. The non-redundant sequences were analyzed with microsatellite finder “Mreps” (http://bioinfo.lifl.fr/mreps/mreps.php) and “Gramene” (http://archive.gramene.org) online protocols to identify sequences containing microsatellite motifs. It is common to find all repeat motifs between mono to hexa nucleotide repeats in plant genome. However, we specifically considered five types of microsatellite combinations with a minimum motif length of 15 nucleotides viz., di nucleotide repeats (DNR), tri nucleotide repeats (TNR), tetra nucleotide repeats (TtNR), penta nucleotide repeats (PNR) and hexa nucleotide repeats (HNR). Primers were designed only for the sequences with repeat motifs between DNR and HNRs using Primer3 algorithm [12]. The quality of primers was determined using the FastPCR (V.5.2.102) program and only those primers that would amplify a fragment in the range of 150 and 500 base pairs of template DNA were selected and custom synthesized. Each of the primer pairs was standardized for their locus specific amplification using the genomic DNA of mulberry genotype, Dudia white as a template. Gradient PCR was carried out in a total reaction volume of 15μL reaction mix containing 25ng of template DNA, 1X Taq buffer, 2mM MgCl_2_, 0.2mM dNTPs, 1U Taq DNA polymerase (MBI, Fermentas Life Sciences, USA) and 3μM each of forward and reverse primers. Amplification was performed in a epGradient Master cycler (Eppendorf, Hamburg, Germany) with the following PCR conditions: denaturation at 95°C for 5 min. followed by 35 cycles of 95°C for 1 min., primer annealing temperatures ranging between 45–65°C for 45 s (depending on the Tm for each primer pair) and an extension step of 72°C for 45 s followed by a final extension step of 72°C for 8 minute. The amplified products were resolved on a 3% (w/v) agarose gel. Only those primer pairs that produced unambiguous single locus amplification were considered for marker development. This stringency ensured the development of robust microsatellite markers in mulberry which can be effectively used for diversity analysis as well as for constructing genetic linkage maps.

### Gene function of the selected EST-SSR sequences

The selected EST sequences were annotated using Blast2GO programme ver.3.3.5 (http://www.last2go.com/) against the public database NCBI for the gene function prediction.

### Validation of genic SSRs

The locus specificity of the markers was examined in three mulberry species, nine varieties and 13 genotypes. These 25 accessions were selected from a set of 1100 germplasm accessions from the Central Sericultural Germplasm Resources Centre (CSGRC), Hosur, Tamil Nadu and Department of Sericulture, University of Agricultural Sciences (UAS), GKVK, Bengaluru, India based on the available passport report information [1]. The list of the mulberry species and genotypes are given in Table 1. Genomic DNA was extracted from young leaf tissues using Amnion mini prep kit (Amnion Biosciences, Bengaluru, India). The template DNA from these mulberry species and genotypes were amplified using the identified locus specific primers. The PCR amplification was carried out with the appropriate annealing temperature standardized by gradient PCR as previously described. The amplified products were analyzed using a microchip based electrophoresis system MultiNA (Shimadzu biotech, Japan). The highest peak detected by the fragment analyzer was scored for the presence of the expected band for each primer pair. The polymorphism data was used to determine the polymorphic information content (PIC), observed heterozygosity and allele diversity for each marker as per Liu and Muse (2005) [13].

**Table 1:** List of mulberry germplasm accessions and species along with other closely related species used for validating the EST-SSR markers developed in mulberry.

**Sl. No.**	**Acc. Name**	**Common name**	**Category**
1	MI-050	*Macrora*	Species
2	ME-08	*Morus Nigra*	Species
3	ME-107	*Morus Lhousering*	Species
4	MI-232	T-12	Genotype
5	MI-240	C-1725	Genotype
6	MI-213	T-21	Genotype
7	ME-03	Moulai	Genotype
8	MI-233	T-08	Genotype
9	MI-0158	C-776	Genotype
10	MI-11	ACC-118	Genotype
11	MI-32	ACC-115	Genotype
12	MI-139	Gajapathipura	Genotype
13	ME-143	SRDC-1	Genotype
14	MI-491	Sabbawala-2	Genotype
15	-	Dudia white	Genotype
16	ME-27	Seizuro	Genotype
17	MI-0052	Mysore local	Variety
18	MI-014	Kanva-2	Variety
19	ME-65	S-1	Variety
20	MI-66	RF-175	Variety
21	MI-0049	S-54	Variety
22	ME-0046	S-13	Variety
23	MI-0021	DD-1	Variety
24	MI-0308	V-1	Variety
25	MI-0012	S-36	Variety
	**Scientific Name**	**Common name**	**Category**
1	*Ficus bengalansis*	fig	Related spp.
2	*Ficus carica*	ficus	Related spp.
3	*Artocarpus heterophyllus*	jackfruit	Related spp.

(Note: All species belong to family *Moraceae*).

### Genetic diversity analysis and transferability to closely related species

It is well known that the genic regions are highly conserved and there would be a significant possibility of a specific microsatellite marker detecting a similar locus in closely related species especially while screening germplasm collections. Establishment of the transferability of markers to other related species is therefore important while developing locus specific marker systems. The transferability of these markers was examined in three closely related species belonging to the family *Moraceae*, namely ficus (*Ficus bengalensis*), fig (*Ficus carica*) and jackfruit (*Artocarpus heterophyllus*). The genomic DNA of these species was extracted using Amnion mini prep kit (Amnion Bioscience, Bengaluru, India).

The percentage of transferability of the markers was calculated using Power Marker statistical tool [13] for each species by determining the presence of target loci to the total number of loci analyzed. The allelic diversity data obtained for all the microsatellite loci amplified were used to compute the genetic dissimilarity using DARwin v.5.0 program [14]. The dissimilarity matrix was further used to generate dendrogram based on UPGMA (Unweighted Pair Group Method with Arithmetic mean) algorithm. This analysis was done to classify the mulberry genotypes/species/other related species based on molecular diversity.

### Construction of genetic linkage map

The SSRs developed in this study (264) along with previously developed SSR markers (487) were screened for polymorphism between the parental lines, Dudia white and UP105. Based on the phenotypic results of an earlier experiment, these genotypes were identified as the most contrasting lines for root traits [15]. A F1 mapping population consisting 150 individuals was developed by using these genotypes. The polymorphic markers were further used for genotyping this F1 population. The amplified products with sequence variation of more than 20 nucleotides were resolved on 3% agarose gel and products with less than 20 nucleotides were screened using a microchip based electrophoresis system (MultiNA, Shimatdzu, Japan). A genotyping data matrix was generated by scoring the banding pattern observed as ‘1’ representing Dudia white and '2’ representing UP105 and ‘3’ representing the heterozygote. Primers with higher product size variability were multiplexed to increase the throughput of fragment analysis. The matrix generated was integrated with the markers and used as an input file in Mapmaker ver.3 for the construction of a framework linkage map. Chi-square test (p <0.05) was performed to assess the aberrant segregation markers in the population and the linkage map was constructed. Linkages between markers were calculated based on logarithm of odds.

## Results

### Frequency and distribution of EST-SSRs

**Fig 1:**
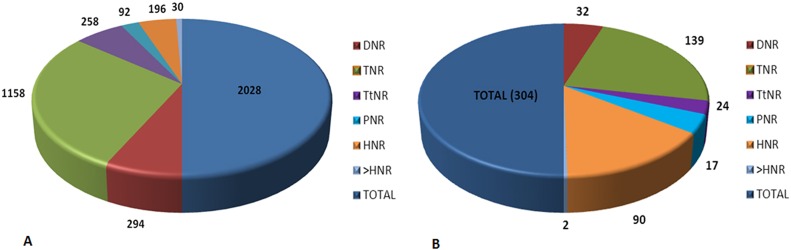
Characterization of EST-SSR and their classification based on repeat motifs. (A) Microsatellite diversity among the EST sequences (B) Microsatellite diversity among the SSR markers identified. Note: Of the 264 sequences identified to be the most appropriate, 304 repeat motifs were identified.

The cDNA library developed from drought stressed leaf tissues of mulberry genotype, Dudia white, contains 10190 ESTs (NCBI-SRA SRP047446). Initially, the ESTs were searched at NCBI against nr database with a stringent E-value and 10169 EST sequences were selected and analyzed for sequence redundancy. A set of 7813 unique ESTs were selected for identifying SSR motifs. Further, this set was analyzed using Mreps and Gramene to discover 1469 sequences with SSR motifs. These sequences were found to harbor 2028 repeat regions among which TNRs were found to be the most abundant accounting for 57% (1158) (Fig 1) followed by 14% of DNR and 13% TtNR motifs. Penta, Hexa and longer than hexa-nucleotide repeats (PNR, HNR and LHNRs) were relatively less frequent among the repeat motifs. However, not all these sequences or repeat motifs could be developed into useful markers. Hence, the criteria for developing scorable SSR markers such as repeat motif size and the total amplicon size were applied while identifying putative sequences. This analysis led to the discovery of 828 sequences harboring 1010 microsatellites (Table 2). These sequences were further examined using the software Primer3 to identify the possible priming regions and manually selected the most appropriate sequences to design primers.

**Table 2:** Characterization of EST library for identification of microsatellite motifs.

Library	Transcripts screened	ESTs with SSR	SSR frequency	SSR with 15 and more nucleotides	Primers developed
EST	10169	1469	2028	1010	264

### Gene function of the selected EST for SSR marker development

**Fig 2:**
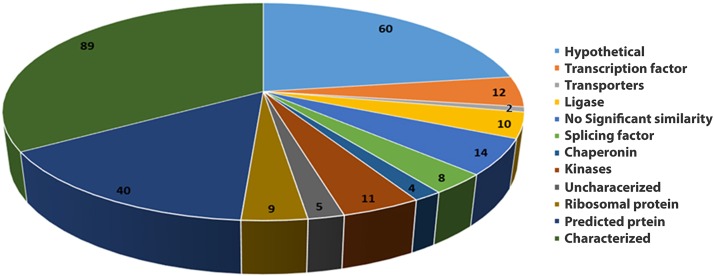
Functional relevance of mulberry EST sequences annotated using NCBI database.

A total of 264 EST sequences satisfied all the criteria for a SSR marker and were selected for marker development. Based on the function, the genes were grouped into different categories like characterized (all sequences with known function), uncharacterized, hypothetical, predicted and sequences with no similarity. About 53% (145) of the EST sequences analyzed had well-defined function that includes major classes such as transcription factors, transporters, ligases, splicing factors, chaperonins, kinases, and ribosomal proteins (Fig 2). The hypothetical and predicted proteins were the other major portions of the selected EST sequences that occupied 24% and 16%, respectively. Around 6% EST sequences did not show any similarity with the existing database sequences and only 1% was uncharacterized sequences (Fig 2).

### Characterization of microsatellite motifs

For developing the markers, MNRs and LHNRs were omitted and the diversity of the repeat types was analyzed. Of the 1010 repeat motifs, 13.8% were found to be DNRs. TNR were the most abundant repeat types accounting for around 50% of the repeat types followed by HNR (19.4%). A mere 5 and 9% of the repeat types were TtNR and PNR, respectively (Table 3). Based on this analysis, a total of 264 sequences were found to be most appropriate, these had the potential for developing into markers and hence primers were designed only for these sequences. Of these 264 sequences, the DNRs TC/AG was found to be most abundant followed by GA/CT and AG/TC (Table 3). The TNRs had 57 diverse motif types. However, among these, GAA, AGA and TCT motifs represented the most frequent TNR repeat types. Motifs like, AAC, ACG and ACT were the least frequent TNRs in the sequences. The TtNR and PNR repeats had 36 and 67 diverse motif types, while HNR were the most diverse with 170 motif variants. The criteria considered for SSR identification revealed several repeat regions with significantly long stretch of motifs. For instance, the GA motif had a repeat length of 70 nucleotides among DNR while “TCT” was the lengthier among TNRs. Among the TtNR and HNRs “AAAT” and “CTCCTT” were the most abundant with a repeat length of 42 nucleotides each.

**Table 3:** Diversity of the repeat motifs among the microsatellite harboring EST sequences.

Contents	DNR	TNR	TtNR	PNR	HNR
**Total no of motifs**	294	1158	258	92	196
**No of motifs (>15Nt)**	140 (13.8%)	501 (50%)	51 (5%)	92 (9%)	196 (19.4%)
**Motif variants**	9	57	36	67	170
**Motifs in 264 Markers**	39	139	17	22	92
**Most abundant motif**	GA, TC, AG	GAA, AGA, TCT	AAAT, ATTT	TTTTA, AAAAT	TTTTTA, CTCCAC
**Least abundant motif**	CA, AC	AAC, ACG, ACT	AAGG, AATA	AAAAC, AAACG	AAAAC, AAATAA

(Note: There were 30 LHNR motifs among the sequences which were not considered in this analysis).

**Fig 3:**
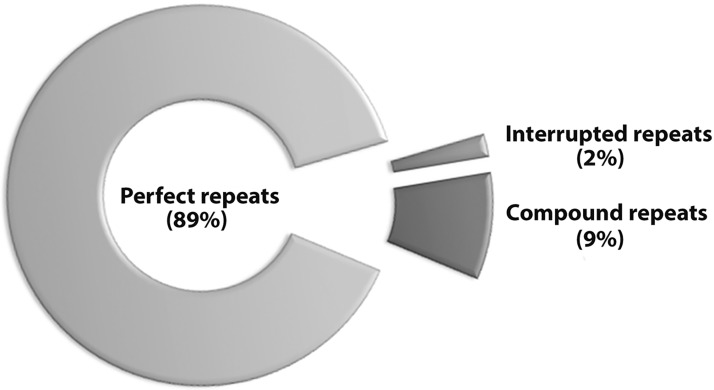
Classification of microsatellite motifs and their frequency among the mulberry EST sequences.

The microsatellite motifs were classified as perfect (presence of similar type of repeat motifs), compound (more than one repeat motif per sequence) and interrupted (repeat motif interrupted by few random bases) based on its arrangement in the EST sequences. Of the 2028 repeat motifs, 89% were found to be perfect repeat type. The compound and the interrupted types accounted 9 and 2%, respectively (Fig 3). The details of the repeat types and their frequency are given in the S1 Table.

### Development of genic SSRs and their validation

After standardizing the annealing temperature for each of these markers, they were screened for locus specific amplification using the genomic DNA of Dudia white. Of all the primer pairs screened, 206 (92%) showed locus specific amplification of the Dudia white genome and were unambiguous indicating the high efficiency of identifying markers by this strategy (Table 4).

**Table 4:** Validation of genic SSR markers in diverse mulberry accessions.

Library	ESTs screened	Markers developed	Locus specific markers	Monomorphic in *Morus* species	Polymorphic in *Morus* species
EST	10169	264	206	17	189 (92%)

**Fig 4:**
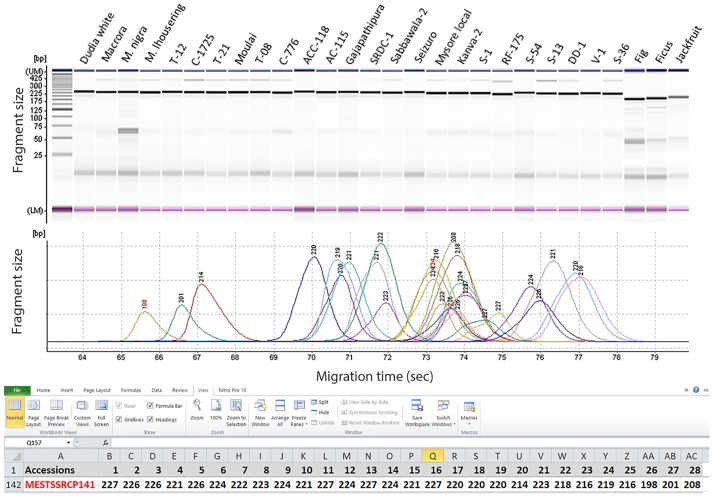
Validation of EST-SSR markers in diverse accession belongs to *Moraceae*. Note: The representative electropherogram showing the amplification profile and scoring of *Moraceae* accessions screened in a capillary electrophoresis system.

To validate the markers, the 206 unambiguous markers were used to screen for the amplification of the genomic DNA of a set of 25 diverse mulberry accessions and species which were selected based on the available passport information to represent a large diversity [1] (Table 1; Fig 4). More than 90% of the 206 locus specific markers displayed polymorphism among the mulberry accessions and species and amplified a total of 620 loci. These markers revealed an average allelic diversity of 3.5 per marker locus which ranged between 1 and 8 alleles per marker locus among all the accessions/species screened. The markers were effective in detecting heterozygosity among the mulberry species which ranged between 0 and 1 with a mean of 0.56. The computed mean PIC value among the species was 0.743. These markers revealed a mean heterozygosity of 0.154 among mulberry genotypes and varieties ranging between 0 and 1. The markers also revealed a higher PIC value of 0.824 and genetic diversity of 84% among the mulberry accessions which were more than that of the mulberry species (Table 5). The results indicated that the genic markers developed can be effectively used for both genetic diversity studies as well as for genetic mapping and QTL discovery applications in mulberry.

**Table 5:** Genetic diversity and polymorphic information revealed by markers developed for mulberry and other closely related species.

Accessions	Range	Genetic diversity (%)	Allele frequency	Heterozygosity	PIC
Mulberry species	Min	27.7	1	0	0
	Mean	61.1	3	0.563	0.332
	Max	77.7	5	1	0.743
Mulberry genotypes/varieties	Min	8.6	1	0	0
	Mean	52.8	1.2	0.154	0.506
	Max	84.4	8	1	0.824

### Trans-species transferability of EST-SSR markers

**Fig 5:**
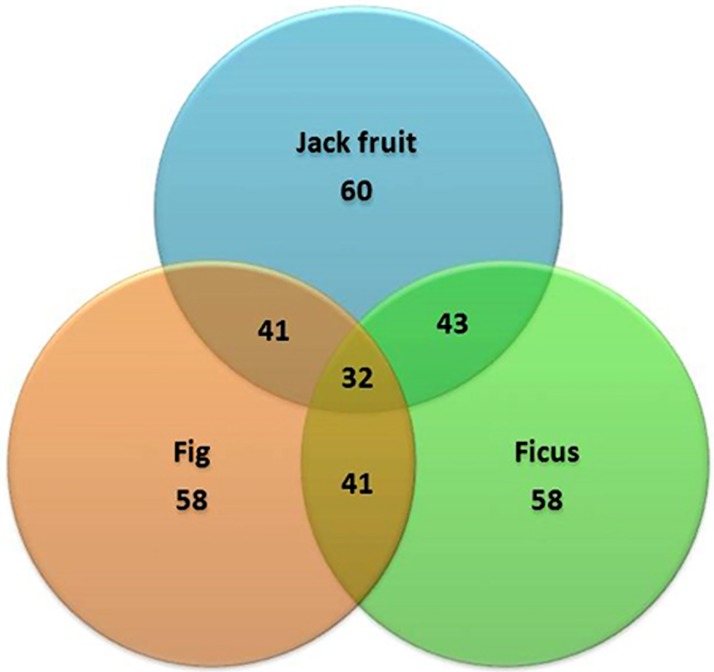
Transferability and distribution pattern of mulberry EST-SSR markers in closely related species belonging to *Moraceae*.

The usefulness of a locus specific SSR marker would be higher if it is transferable to other closely related species. We assessed the transferability of the mulberry genic SSR markers using three species belonging to the family *Moraceae*, namely fig, ficus and jackfruit. Of the 206 markers developed, 40% (83) were transferable to these closely related species (Table 6). The number of markers that were specific to a particular species and those shared between pairs of species and all the three species is illustrated in Fig 5. The PIC value revealed by these markers ranged between 0 and 0.743 with a mean of 0.666 and they detected a higher heterozygosity of 0.77 among the species (Table 7). The genetic diversity revealed by these markers among the other species of *Moraceae* was also high ranging from 27 to 77% with a mean of 68%.

**Table 6:** Transferability of mulberry genic SSRs in three closely related species belonging to *Moraceae*.

Library	Locus specific markers	Transferable markers	Transferability		
			Fig	Ficus	Jackfruit
EST	206	83 (40.2%)	58 (28%)	58 (28%)	60 (29%)

**Table 7:** Genetic diversity and polymorphic information revealed by mulberry EST-SSR markers in fig, ficus and jackfruit.

Accessions	Range	Genetic diversity (%)	Allele frequency	Heterozygosity	PIC
Other related species	Min	27.7	1	0	0
	Mean	68.3	1.15	0.13	0.666
	Max	77	5	0.77	0.743

### Genetic diversity

**Fig 6:**
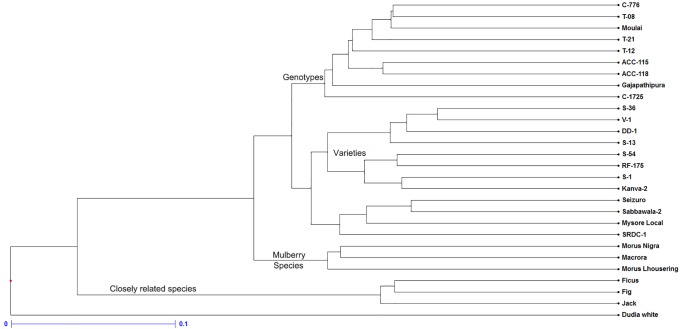
Diversity analysis of mulberry and closely related species belongs to *Moraceae* using 206 EST-SSR markers.

The ability of a marker to distinguish between related and unrelated individuals determines its usefulness of the marker. A neighbor joining algorithm based on UPGMA was adopted to assess the diversity among species and genotypes. The dendrogram illustrated in Fig 6 indicated a clear segregation of mulberry and other species into two distinct clades. Further, the three mulberry species used in this study were grouped into single cluster which was distinctly different from the mulberry genotypes. Since, the genomic DNA of Dudia white was used for marker development, the entire set of markers amplified in Dudia white and hence, this genotype grouped itself into a separate cluster away from the rest of the accessions. Among the accessions screened, the maximum genetic similarity was observed between S-36 and V-1 and least was between Dudia white and Macrora (S2 Table).

### Construction of linkage map

A total of 751 SSR markers including 264 EST-SSRs reported in this study and those reported by us previously (351 genomic and 400 genic) were screened for parental polymorphism. Of these markers, 453 were found to be polymorphic between Dudia white and UP105. The polymorphic loci ranged from 150 to 500 bp with an average of 235 bp were further used to genotype the mapping population. Scoring of the marker profile revealed varying degrees of segregation in the population. The markers are expected to segregate in a 1:1 ratio since the F1 generation allows a maximum recombination percentage of 50 for each parent. Chi-square analysis of goodness-of-fit revealed a total of 262 (57.83%) unambiguous markers that followed a typical test cross segregation ratio (1:1) which were further used for the construction of a genetic linkage map.

**Fig 7:**
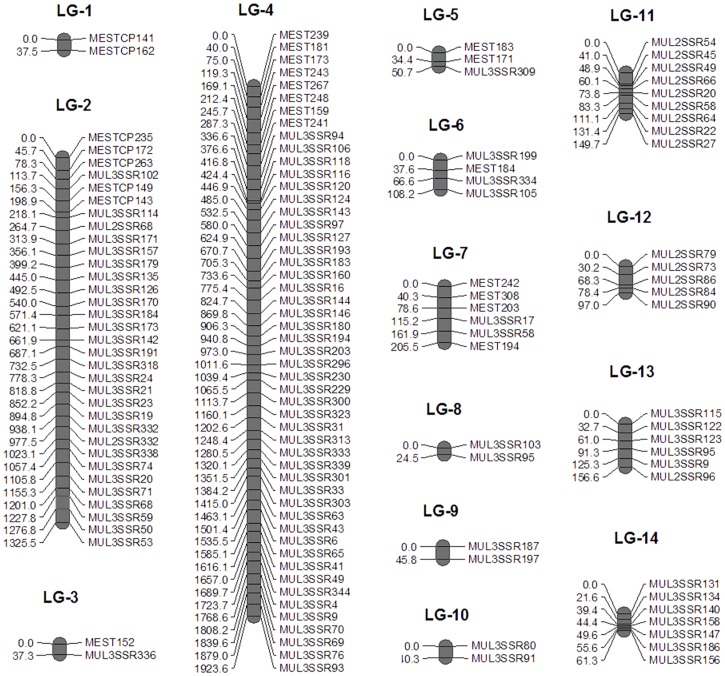
Construction of genetic linkage map in mulberry exclusively using co-dominant markers.

Overall, linkages were robust and a minimum LOD value of 3 was fixed. A total of 134 (111 genomic and 23 genic) markers segregating in 1:1 test ratio could be aligned on the map (Table 8; Fig 7). The markers covered a total map distance of 4263.5 cM on 14 linkage groups. The linkage map with a total of 14 linkage groups matches the karyotype of mulberry (2n = 28). The distribution of the markers was random and unequal between linkage groups and clustered in some regions (LG2 and LG4). The distance between two linked markers varied from 5.0 cM between MUL3SSR140 and MUL3SSR158 to 49.8 cM between MESTSSRCP243 and MESTSSRCP267 on LG4. The average map distance observed between two linked markers was 31.8 cM. The largest linkage group LG4 had map coverage of 1923.6 cM with 51 markers followed by LG2 with 33 markers spanning 1325.5 cM (Table 8; Fig 7).

**Table 8:** Details of the linkage map developed, distance covered and number of linked marker in each linkage groups.

Linkage Group (LG)	Map Length (cM)	Linked markers
LG-1	37.5	2
LG-2	1325.5	33
LG-3	37.3	2
LG-4	1923.6	51
LG-5	50.7	3
LG-6	108.2	4
LG-7	205.5	6
LG-8	24.5	2
LG-9	45.8	2
LG-10	40.3	2
LG-11	149.7	9
LG-12	97	5
LG-13	156.6	6
LG-14	61.3	7
Total	4263.5	134

In all, we developed a total of 206 highly reproducible, unambiguous genic SSR markers in mulberry. These markers will be extremely useful for phylogenetic and evolutionary studies besides being highly useful in developing linkage maps and identifying genomic regions governing complex agronomic traits in mulberry. A significant portion of these markers was transferable to other closely related species, which paves way for deploying these markers in diversity analysis in these species as well.

## Discussion

Production of quality mulberry leaves is pivotal for achieving raw silk production targets [2]. Although mulberry cultivation is possible in diverse environments, this crop species is quite sensitive to change in environmental variables, especially water [16]. Water limitation even in irrigated regions can also cause significant loss in yield and quality of mulberry leaves [17]. Hence, developing effective strategies to enhance the yield potential and stress resilience has paramount significance. Being a heterozygous and out-breeding perennial species, genetic enhancement of mulberry has remained a challenge necessitating the adoption of modern genomics based breeding approaches of crop improvement [18]. Availability of DNA based molecular markers is therefore a prerequisite for initiating such molecular breeding programs. Towards this, we recently reported a set of genomic SSR markers for mulberry [7]. However, this report comprised predominantly of SSR markers identified in the non-coding regions of the mulberry genome. It is well recognized that variations in the coding regions can lead to the identification of functional markers linked with specific traits of interest. Towards enriching this repository of SSR markers, a set of EST database comprising of over 10000 sequences were analyzed (NCBI-SRA SRP047446) [10] to identify 1469 ESTs which harbored 2028 microsatellite regions. For a repeat motif to be developed into a marker, the repeat motif should be ≥15 bp with a total amplicon size of ≥150 bp [19]. Only 1010 ESTs satisfied these criteria, which represented only 12% of the ESTs analyzed after eliminating the redundancy. It is apparent that the events causing the microsatellite motif like replication slippage, cross over and point mutations are less likely to happen in genic regions than in the non-coding regions of the genome [19][20]. On the contrary, we reported earlier that SSR markers were more abundant in the non-coding regions of the genome representing more than 30% [7].

Among the genic microsatellite motifs identified in the present investigation, TNR types were most frequent representing 50% of the repeat motifs followed by the HNRs (19%). Similarly, high proportions of TNR and HNR have been reported in genic regions in species such as rice [21], sugarcane [22], pea [23] including human being [24]. The presence of higher proportion of TNR in mulberry corroborates the earlier reports of TNR being the main EST-SSR repeat type in both monocots and dicots [25][26][27]. The presence of tri nucleotide diversity among the expressed sequence could be explained as its tolerance over other nucleotide types for addition and deletion during cell cycles. The TNR and HNRs found in mulberry were predominately AT rich; such AT rich repeats in the TNRs was reported in *Arabidopsis* and yeast genomes [28][29]. Among the 57 diverse TNR identified, “GAA”, “AGA” and “TCT” were found to be the most frequent.

Among the DNRs, “TC/AG” repeats constituted the most frequent DNR motifs followed “GA/CT” in ESTs, while our earlier report on genomic SSRs also consisted of the same set of microsatellite variants [7]. Occurrence of such microsatellites in both genic and genomic region was reported in other crop species like peanut [30], and also in the human genome [24]. Even the TtNRs, the microsatellite motifs were AT rich with “AAAT” motifs being the most abundant; which is similar to our earlier report on genomic SSRs [7]. The presence of such similar repeat types in both genic and genomic regions show their dominance in occurrence in the mulberry genome.

We characterized the microsatellite motifs based on the repeat sequence frequency into perfect, interrupted and compound SSRs. Majority of the repeat motifs were of the perfect sort (89%). A similar trend was also observed in SSR motifs of non-coding regions [7]. The frequency of microsatellite occurrence among the mulberry transcriptome sequences screened was one locus per 1.44 kb, which is significantly higher than other crop species like soybean (7.4kb), wheat (5.4kb) and coffee (3.44kb) [28][31][32]. This perhaps reveals the occurrence of higher sequence rearrangements in mulberry. Nevertheless, the frequency of occurrence of microsatellite regions is less in genic regions than the non-coding regions [19][20].

In our previous report [7] markers were developed from global transcript data from a mulberry cultivar K-2 [7]. The marker information present in this investigation were generated from the transcriptome developed from drought stressed accession of the germplasm namely Dudia white. These two transcriptomes were expected to be mutually exclusive because of the genetic difference as well as the conditions of the plant growth. To verify this, the extent of redundancy between the two data sets was compared. We found one EST namely MESTSSRCP-140 was exactly same as that of MESTSSR-121 in the previous study. Functional annotation of this EST sequence revealed no specific function and the analysis returned a hypothetical protein located on *Morus notabilis* genome. This redundant marker is highlighted in the S1 Table.

### Validation of EST-SSR markers

EST-SSRs have emerged as an efficient marker system for genetic mapping, studying phylogenetic relationships and functional diversity among closely and also in distantly related accessions. Their competence in identifying polymorphism and hence diversity among related species have been verified in different crop species [33][34]. The genic SSRs, though less abundant, have an additional possibility of qualifying to be functional markers [35].

Two approaches were adopted in this study to validate the EST-SSRs. We used mulberry species and genotypes (including germplasm and cultivated varieties) in the first approach and in the second approach, a few species belonging to the same family *Moraceae*, viz., fig, ficus and jackfruit were used. This strategy enabled the validation of genic SSR markers besides generating useful information on the transferability of these markers to other related species. Out of the 264 perfect primer pairs developed for genic microsatellite regions, we validated a set of 206 markers using the Dudia white genomic DNA. While all the 206 genic SSRs amplified a single locus in all mulberry species and genotypes, 92% of them (189) revealed polymorphism among mulberry accessions. Higher proportion of polymorphism explained by EST markers was reported in other plant species like *Medicago* [33], *Citrus* [36], *Amorphophallus* [37] and *Chrysanthemum* [38]. The primers were developed at the exonic regions of cDNA which might recognize the intron length variations. This could be one of the reasons for a higher level of polymorphism of EST-SSR markers. This polymorphism resembles the intron length polymorphism (ILP) which is recently being exploited as a powerful marker system [39].

The variability obtained for the resolved markers among the mulberry accessions was analyzed using Power Marker version 3.25. A total of 620 alleles identified by 206 markers ranged between 1 and 8 with an average of 3.5 alleles per marker locus. However, this was less compared to the allelic diversity reported in non-coding region of mulberry [7]. This could be because of greater tolerance of genic region to genome shuffling and rearrangement than genomic regions that are commonly prone to such alterations [20]. A few of the markers (ESTSSRCP-212, ESTSSRCP-213, ESTSSRCP-226, ESTSSRCP-239 and ESTSSRCP-396) were able to identify private alleles. Such markers are useful in affirming genetic identity of a particular accession in germplasm characterization [40]. The efficiency of any marker system irrespective of its source is determined by its ability to capture the polymorphic information content. The PIC value observed was high for most of the markers with the maximum of 0.824 among mulberry accessions and 0.743 among mulberry species (Table 5). Similarly, the markers were efficient in identifying heterozygosity in the range of 0 to 1 among the accessions and species which is important for a cross pollinating highly heterozygous crop like mulberry. The higher genetic diversity explained by these markers among the accessions and species substantiate their efficiency as effective markers for diversity studies which can be extended to QTL mapping of complex traits.

### Transferability of the genic SSR markers to other related species

Examining the transferability of the mulberry genic SSRs to other related species is considered as a robust validation of markers besides providing greater value to the markers developed in diversity analysis. We examined the transferability of all the markers developed to a few species like fig, ficus and jackfruit belonging to the family *Moraceae* that are close relatives of mulberry [34]. A total of 60 (29%), 58 (28%) and another 58 (29%) markers amplified the genomic DNA of jackfruit, fig and ficus, respectively, with 40% of all markers transferable to these species (Table 6). Evolutionarily, species belonging to a specific genus or family have certain relatedness at the genomic level. It is therefore, plausible that the markers are transferable across these species belonging to the same family. Further, it is apparent that the coding regions are more conserved across the phyla than the non-coding parts of the genome. Accordingly, more percentage of the genic SSRs were transferable to other related species than what we noticed for the SSR markers developed for the non-coding regions of mulberry genome [7]. All these transferable markers were informative with PIC value ranging up to 0.743 and maximum heterozygosity value of 0.77. Considering the inadequate availability of informative markers in this commercial species, this study provided a strong impetus by developing co-dominant EST-SSR markers. The molecular diversity among germplasm as well as across wild species can be effectively documented using EST-SSR markers [35]. The three mulberry species examined in this study showed an average of 70% similarity, which is particularly useful while considering these species in hybridization programmes. It is apparent that sequence variations in the coding regions of the genome could be minimal. Differences, if at all detected, the EST-SSR markers would be an excellent tool to ascribe the functional diversity at genic level.

The EST-SSR markers successfully segregated the genotypes from the mulberry species. Further, the other related species were grouped into a separate cluster. Among the genotypes S-36 and V-1 showed greater genetic relatedness (80% similarity) while Dudia white and Macrora were the most distant pair (13% similarity). However, the diversity observed among the accessions using the genic markers were moderate compared to the genomic markers used in our earlier report which confirms the higher conservative nature of genic markers. Therefore, the genic SSR markers have the advantage in studying the functional variations of mulberry accessions and related species of *Moraceae*.

### Linkage mapping in mulberry

Besides the diversity analysis, the other major applications of the markers is in developing genetic linkage maps. Absence of extensive genomic resources and complexity arising due to the heterozygosity of the genome severely hinders the construction of genetic linkage maps in this out breeding perennial species. Although, skeletal maps have been reported [41][42] most of the markers used in these studies were dominant markers. The set of genomic and genic SSR markers reported from our group earlier [7] and in this study, represents useful genomic resource of co-dominant markers. The F1 full-sib progeny that mimic the original F2 population was used to construct a genetic linkage map adopting a “pseudo-test-cross” approach [43]. In this study, similar mating configuration was employed to analyze segregation of markers in the F1 population of Dudia White and UP105 genotypes.

Microsatellite markers are very useful tools for both creating and expanding linkage maps. They are capable of detecting variation in both the alleles of a specific locus and facilitates in precise mapping even in heterozygous organisms. The present study is an exclusive report on constructing linkage map using SSR markers only. Of the total 751 markers developed, 453 were polymorphic between the parental lines. Chi-square test was performed to test the 1:1 segregation ratio of the markers and 262 such markers were further used for map construction. The markers deviating from the expected segregating ratio were excluded from the map because they share insufficient information for linkage analysis and increase the chance of inaccurate and false linkage.

The genic markers developed in the present study showed more segregation distortion compared to genomic markers. Linkages between markers were calculated using odds ratio. Reasonable genome coverage was achieved with this mapping strategy. An exact number of linkage groups in relation to the haploid chromosome number of mulberry (n = 14) was observed. The number of markers linked in each group varied between two to 52. Uneven distribution of markers in the map is a common phenomenon because the frequency of crossing over or recombination events is not necessarily equal in all the linkage groups [44][45]. The absence of the clustering of markers in some linkage group may be due to suppression of recombination around the centromere region suggesting that mulberry is naturally resistant to such genetic effects similar to those reported in intraspecific crosses of tree species [46].

In this study we report development of 751 co-dominant markers for mulberry. Besides being extensively useful in diversity analysis, these markers can be effectively used for identifying specific genomic regions that govern the variability in complex quantitatively inherited traits. The framework linkage map developed paves way for future QTL mapping.

## Conclusion

Genomics assisted breeding has emerged as the most appropriate and powerful strategy in accelerating crop improvement. Non availability of robust marker systems had been the major limitation in adopting genomics based breeding strategies in mulberry. This lacuna has now been overcome to a certain extent with the reporting of 751 SSR markers. This co-dominant marker system that can recognize heterozygosity would strongly enhance the effectiveness of determining genetic/molecular diversity, especially in the out breeding, heterozygous species like mulberry. Having been developed from expressed regions of the mulberry genome, these markers would be extremely useful in developing functional markers to aid the crop improvement. Mapping QTL for specific drought adaptive traits using these markers is under way.

## Supporting Information

S1 Table Details of EST-SSR markers developed for mulberry. (XLSX)

S2 Table Dissimilarity matrix of mulberry and other related species tested for transferability of genic and genomic SSR markers. (XLSX)
